# Related factor analysis for predicting large-volume central cervical lymph node metastasis in papillary thyroid carcinoma

**DOI:** 10.3389/fendo.2022.935559

**Published:** 2022-08-15

**Authors:** Li Tan, Jiaqi Ji, Gaowa Sharen, Yuewu Liu, Ke Lv

**Affiliations:** ^1^ Department of Ultrasound, Peking Union Medical College Hospital, Chinese Academy of Medical Sciences and Peking Union Medical College, Beijing, China; ^2^ Department of Ultrasound, Aerospace Center Hospital, Beijing, China; ^3^ Department of Health Management, Peking Union Medical College Hospital, Chinese Academy of Medical Sciences and Peking Union Medical College, Beijing, China; ^4^ Department of General Surgery, Peking Union Medical College Hospital, Chinese Academy of Medical Sciences and Peking Union Medical College, Beijing, China

**Keywords:** papillary thyroid carcinoma, ultrasound, lymph node metastases, thyroid cancer, cervical lymph node metastases

## Abstract

The aim of this study was to investigate the factors related to large-volume central cervical lymph node metastasis (LNM) in papillary thyroid carcinoma. A retrospective study of 340 patients with 642 papillary thyroid carcinoma nodules who underwent thyroidectomy in Peking Union Medical College Hospital between 2011 and 2015 was conducted. These nodules were divided into two groups by the number of central cervical lymph node metastases: large‐volume central cervical LNM (>5 metastatic lymph nodes, *n* = 129) and no central cervical LNM (*n* = 211). We evaluated the correlations between gender, age, chronic lymphocytic thyroiditis, thyroid ultrasonographic features, and large‐volume central cervical LNM. We found that younger age (≤40 years) (OR = 3.796, 95% CI = 2.842, 5.070), male gender (OR = 4.005, 95% CI = 2.858, 5.61), and ultrasonographic features such as tumor macroaxis size (OR = 2.985, 95% CI = 1.581, 5.633), tumor located in the isthmus (OR = 7.578, 95% CI = 4.863, 11.810), ill-defined margin (OR = 3.008, 95% CI = 1.986, 4.556), microcalcification (OR = 2.155, 95% CI = 1.585, 2.929), and abnormal cervical lymph nodes (OR = 13.753, 95% CI = 9.278, 20.385) were independent risk factors for large-volume central cervical LNM in papillary thyroid carcinoma, while chronic lymphocytic thyroiditis (OR = 0.248, 95% CI = 0.172, 0.358) was a protective factor. Younger age (≤40 years), male sex, and ultrasonographic features such as tumor macroaxis size, tumor located in the isthmus, ill-defined margin, microcalcification, and abnormal cervical lymph nodes were independent risk factors for large-volume central cervical LNM in papillary thyroid carcinoma, while chronic lymphocytic thyroiditis can be considered a protective factor. Our results provide a reference for adjusting clinical treatment approaches.

## Introduction

The incidence of thyroid cancer is increasing globally (1). Papillary thyroid carcinoma (PTC) is the most common kind of thyroid cancer. PTC is associated with high morbidity, low mortality, and favorable prognosis; however, it has been reported that large-volume cervical lymph node metastasis (LNM) is associated with higher recurrence rates and poor prognosis (2–4). The 2015 American Thyroid Association (ATA) guidelines updated the risk stratification of recurrence, including large-volume cervical LNM (>5 metastatic lymph nodes) as a medium-risk factor for recurrence (4). The central region of the neck is the most common area of LNM in PTC, while the preoperative detection rate is low. Furthermore, the optimal treatment strategy for thyroid malignancy is controversial, and opinions are divided on whether central compartment lymph nodes (LNs) in patients without LNM detected by preoperative ultrasound require dissection. Therefore, there is an urgent need to identify related factors that may predict the metastasis potential of PTC, which is crucial for informing clinical therapy approaches. We conducted a retrospective study to analyze the clinical characteristics and ultrasound images of PTC nodules and summarized the factors related to large-volume central cervical LNM (CCLNM) to support clinical decision making and optimize treatment effectiveness.

## Materials and methods

### Patients and definitions

A total of 340 patients including 64 men and 276 women with pathologically proven PTC who underwent surgery in Peking Union Medical College Hospital between 2011 and 2015 were enrolled.

**Figure 1:**
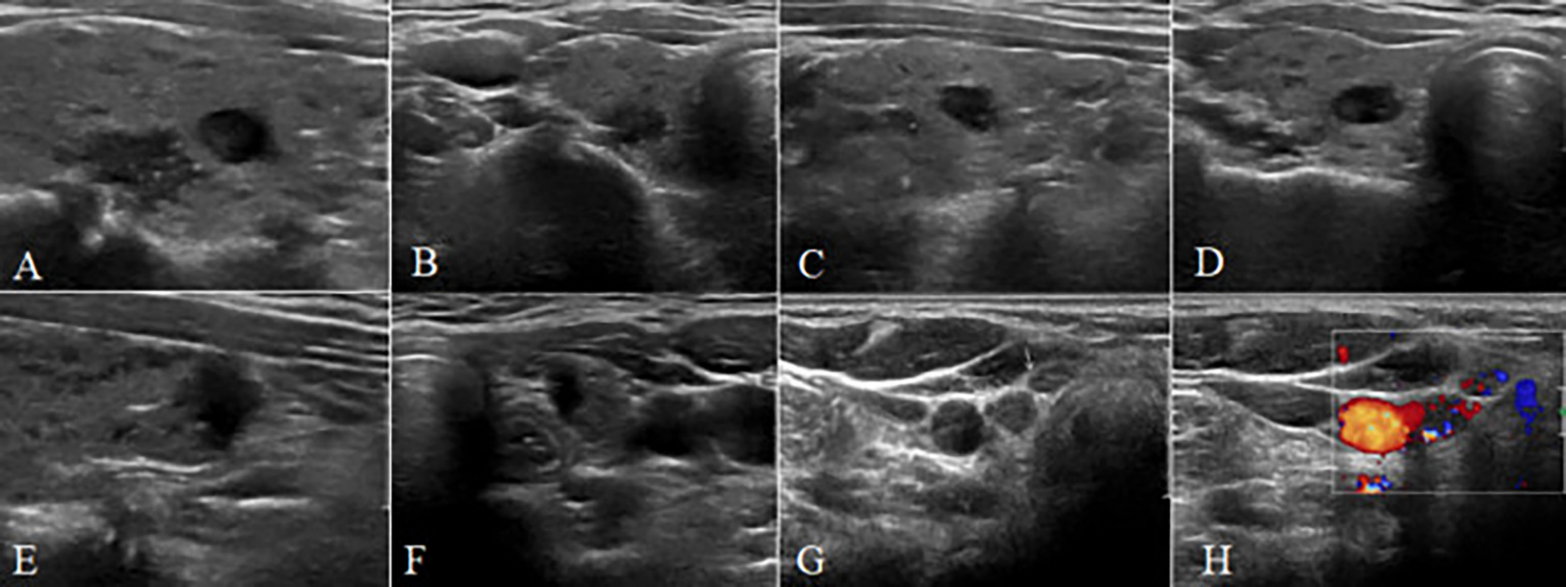
A 38-year-old female patient with multiple papillary thyroid carcinoma and large-volume CCLNM. **(A–F)** The longitudinal and transverse gray-scale sonogram of the tumor. **(A, B)** A bilateral nodule (11 mm × 9 mm × 6 mm) with irregular shape, spiculated margin, A/T < 1, close to capsule, away from trachea, and microcalcification. **(C, D)** A bilateral nodule (7 mm × 8 mm × 5 mm) with irregular shape, spiculated margin, A/T < 1, away from capsule, and away from trachea. **(E, F)** A bilateral nodule (8 mm × 5 mm × 8 mm) with irregular shape, ill-defined margin, A/T > 1, capsule interruption, and close to trachea. **(G)** The gray-scale sonogram of central lymph nodes showing round-like nodes without normal structure (arrows). **(H)** The color sonogram of central lymph nodes showing abundant blood supply with irregular distribution. CCLNM, central cervical lymph node metastasis.

The inclusion criteria were as follows. 1) The patient underwent his/her first surgery in Peking Union Medical College Hospital and PTC was pathologically confirmed. 2) The scope of surgical resection was at least thyroidectomy and dissection of more than five central compartment LNs on the lesion side. 3) Preoperative ultrasonography was performed, and the following observations were made: i) thyroid malignant nodules were recorded: a) location, size, shape, and margin; b) condition of trachea invasion; c) number: single or multiple; d) condition of capsular involvement; e) the ratio of anteroposterior (A) and transverse (T) diameter (A/T ≥ 1 or A/T < 1) of the nodule; f) condition of calcification: no calcification, microcalcifications, or macrocalcifications. Microcalcifications had tiny, punctuated hyperechoic foci <1 mm in diameter, either with or without acoustic shadows. ii) The shape, structure, and blood flow distribution of central cervical LNs were observed to evaluate whether they were normal or abnormal. The following criteria were used for sonographic indicators of abnormal cervical LNs: a) microcalcification in the LN; b) liquefied or cystic appearance in the LN; c) the LN was round or quasi-round, with A/T < 2; d) loss of lymphatic hilum; e) hyperechoic mass in the LN; f) abundant blood supply, with peripheral vascularization or mixed flow. If a), b), or at least two items of c)–f) were met, the LN was synthetically judged as metastatic (5). Sonographs of PTC patients were evaluated and recorded by two senior physicians (**Figure 1**). 4) The number of metastatic LNs of pathologically confirmed CCLNM was >5, or there was no CCLNM. 5) The clinical data were complete, including the patient’s gender, age, and presence of chronic lymphocytic thyroiditis (CLT).

The exclusion criteria were as follows: 1) a non-pathologic diagnosis of CCLNM was made, or the number of CCLNM did not meet the grouping criteria; 2) preoperative ultrasound images were incomplete; 3) clinical data were incomplete.

The study was approved by the Ethics Committees of Peking Union Medical Hospital, and informed consent was obtained from all patients. We divided PTC nodules into two groups by the number of CCLNM according to pathology: large‐volume CCLNM (>5 metastatic LNs) and no CCLNM.

### Statistical analysis

Categorical data are presented as absolute and relative frequency; continuous data are presented as mean and standard deviation. To compare quantitative variables with qualitative variables, the two-tailed *t*-test for independent samples was used. Logistic regression was used for univariate and multivariate analyses. Statistical significance was defined as *p* < 0.05. All analyses were performed by SPSS19.0.

## Results

In total, 340 patients were pathologically diagnosed with 642 PTC nodules, including 157 cases with large‐volume CCLNM and 305 cases without CCLNM. The basic characteristics of the participants are presented in **Table 1**.

**Table 1:** Basic characteristics of participants.

	Large-volume CCLNM (*n* = 157)	No CCLNM (*n* = 305)	*p*
Age (%)			<0.001
≤40	85 (54.1)	73 (23.9)	
>40	72 (45.9)	232 (76.1)	
			
Gender (%)			<0.001
Male	54 (34.4)	27 (8.9)	
Female	103 (65.6)	278 (91.2)	
			
Ultrasound features			
Location (%)			<0.001
Bilateral	125 (79.6)	223 (73.1)	
Isthmus	32 (20.4)	82 (26.9)	
			
Capsular invasion (%)			0.059
Close to capsule	33 (21.0)	95 (31.1)	
Capsule interruption	13 (8.3)	18 (5.9)	
Away from capsule	111 (70.7)	192 (63.0)	
Trachea involvement (%)			0.568
Away from trachea	151 (96.2)	297 (97.4)	
Close to trachea	6 (3.8)	8 (2.6)	
			
Number (%)			0.157
Single	124 (79.0)	257 (84.3)	
Multiple	33 (21.0)	48 (15.7)	
			
Size (mean ± SD)			<0.001
Macroaxis	1.54 (0.90)	0.92 (0.50)	<0.001
Transverse	0.98 (0.45)	0.69 (0.32)	<0.001
Anteroposterior	1.14 (0.60)	0.74 (0.38)	<0.001
			
Shape (%)			<0.001
Round to oval	54 (34.4)	176 (57.7)	
Irregular	103 (65.6)	129 (42.3)	
			
Margin (%)			<0.001
Smooth	66 (42.0)	182 (59.7)	
Spiculated	58 (36.9)	100 (32.8)	
Ill-defined	33 (21.0)	23 (7.5)	
			
A/T (%)			0.162
A/T < 1	133 (84.7)	242 (79.3)	
A/T ≥ 1	24 (15.3)	63 (20.7)	
			
Calcification (%)			<0.001
No calcification	30 (19.1)	154 (50.5)	
Microcalcification	120 (76.4)	127 (41.6)	
Macrocalcification	7 (4.5)	24 (7.9)	
			
LN (%)			<0.001
Normal	92 (58.6)	293 (96.1)	
Abnormal	65 (41.4)	12 (3.9)	
			
CLT (%)			0.013
Without CLT	134 (85.4)	230 (75.4)	
With CLT	23 (14.6)	75 (24.6)	

CCLNM, central cervical lymph node metastasis; LN, lymph node; CLT, chronic lymphocytic thyroiditis.

As shown in **Table 1**, there were statistically significant differences between the two groups in age, gender, tumor location, tumor size, tumor shape, margin, condition of calcification, the presence of abnormal LNs, and the occurrence of CLT (*p* < 0.05). No significant differences were observed in tumor number, A/T, and the relationship between the tumor and capsule or trachea (*p* > 0.05). Univariate analysis was used to analyze the significant factors (**Table 2**).

**Table 2:** Univariate analysis for large-volume CCLNM.

	OR (95% CI)	*p*
Age		<0.001
>40	1	
≤40	3.752 (3.124, 4.506)	
		
Gender		<0.001
Female	1	
Male	5.131 (2.958, 8.902)	
		
		
Ultrasound features		
Location		<0.001
Bilateral	1	
Isthmus	4.163 (3.029, 5.720)	
		
Size		
Macroaxis	4.480 (3.694, 6.863)	<0.001
Transverse	8.539 (6.300, 11.572)	<0.001
Anteroposterior	7.007 (5.432, 9.039)	<0.001
		
Shape		<0.001
Round to oval	1	
Irregular	2.159 (1.415, 3.294)	
		
Margin		0.001
Smooth	1	
Spiculated	1.306 (0.833, 2.047)	
Ill-defined	3.326 (1.767, 6.259)	
		
Calcification		<0.001
No calcification	1	
Microcalcification	4.167 (2.537, 6.846)	
Macrocalcification	1.284 (0.494, 3.335)	
		
LN		<0.001
Normal	1	
Abnormal	18.057 (8.619, 37.832)	
		
CLT		0.003
Without CLT	1	
With CLT	0.453 (0.268, 0.767)	

CCLNM, central cervical lymph node metastasis; LN, lymph node; CLT, chronic lymphocytic thyroiditis.

In the univariate analysis, we found that age ≤40 years (OR = 3.752, 95% CI = 3.124, 4.506), male gender (OR = 5.131, 95% CI = 2.958, 8.902), tumor located in the isthmus (OR = 4.163, 95% CI = 3.029, 5.720), macroaxis size (OR = 4.480, 95% CI = 3.694, 6.863), transverse size (OR = 8.539, 95% CI = 6.300, 11.572), anteroposterior size (OR = 7.007, 95% CI = 5.432, 9.039), irregular shape (OR = 2.159, 95% CI = 1.415, 3.294), ill-defined margin (OR = 3.326, 95% CI = 1.767, 6.259), microcalcification (OR = 4.167, 95% CI = 2.537, 6.846), and abnormal LNs (OR = 18.057, 95% CI = 8.619, 37.832) were risk factors for large-volume CCLNM, while CLT (OR = 0.453, 95% CI = 0.268, 0.767) was a protective factor. However, a spiculated margin (OR = 1.306, 95% CI = 0.833, 2.047) and macrocalcification (OR = 1.284, 95% CI = 0.494, 3.335) showed no statistical significance in the univariate analysis (**Table 2**). Multivariate analysis was conducted to further analyze the significant factors (**Table 3**).

**Table 3:** Multivariate analysis for large-volume CCLNM.

	OR (95% CI)	*p*
Age		<0.001
>40	1	
≤40	3.796 (2.842, 5.070)	
		
Gender		<0.001
Female	1	
Male	4.005 (2.858, 5.613)	
		
Ultrasound features		
Location		<0.001
Bilateral	1	
Isthmus	7.578 (4.863, 11.810)	
		
Size		
Macroaxis	2.985 (1.581, 5.633)	<0.001
Transverse	1.087 (0.464, 2.546)	0.848
Anteroposterior	1.014 (0.380, 2.705)	0.978
		
Shape		0.760
Round to oval	1	
Irregular	1.045 (0.789, 1.383)	
		
Margin		<0.001
Smooth	1	
Ill-defined	3.008 (1.986, 4.556)	
		
Calcification		<0.001
No calcification	1	
Microcalcification	2.155 (1.585, 2.929)	
		
LN		<0.001
Normal	1	
Abnormal	13.753 (9.278, 20.385)	
		
CLT		<0.001
Without CLT	1	
With CLT	0.248 (0.172, 0.358)	

CCLNM, central cervical lymph node metastasis; LN, lymph node; CLT, chronic lymphocytic thyroiditis.

**Figure 2:**
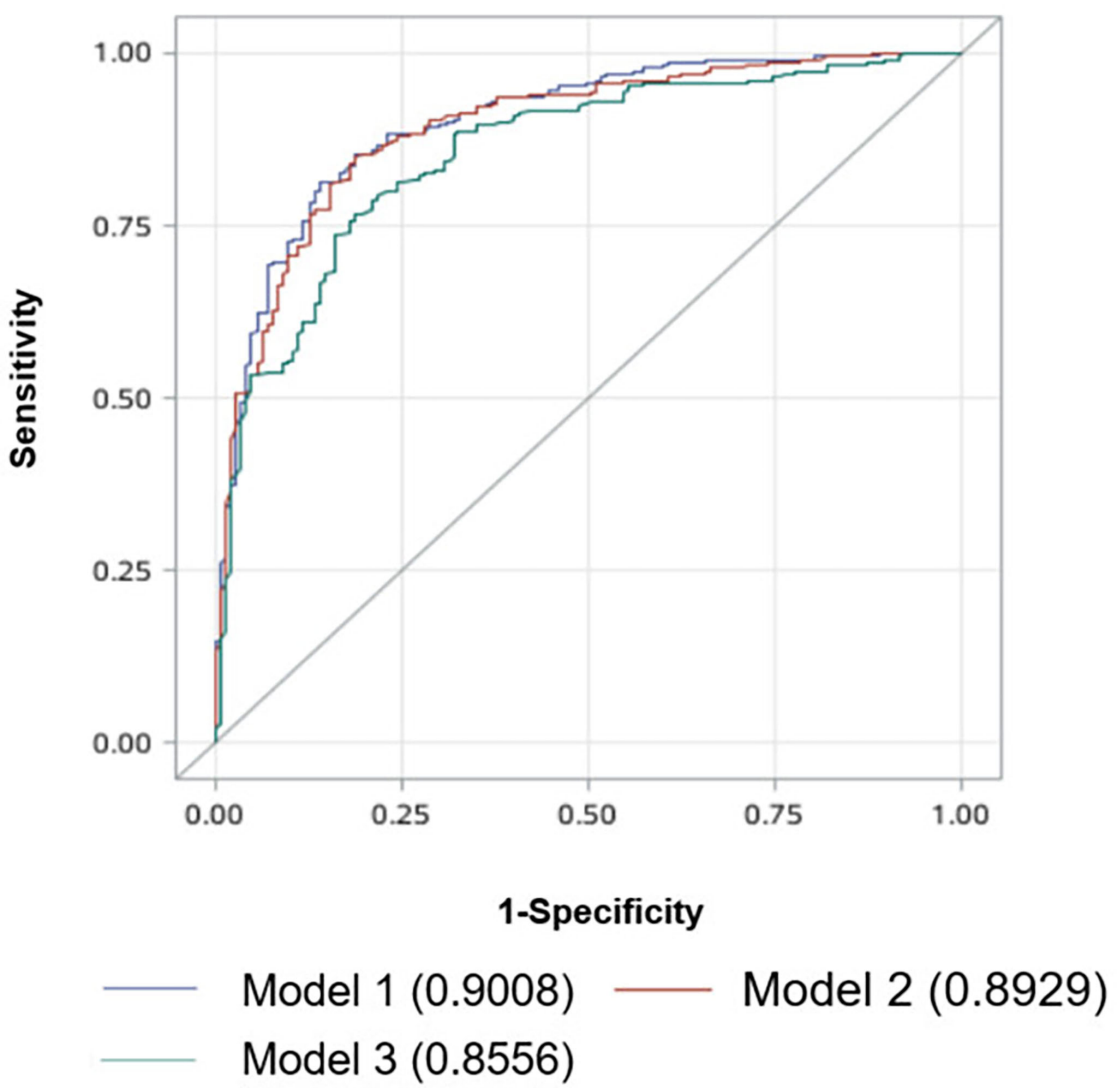
Receiver operating characteristic curve analysis for predicting large-volume CCLNM. Model 1: factors include age, gender, CLT, and ultrasound features such as tumor location, tumor size, margin, calcification, and abnormal LNs. The AUC is 0.9008. Model 2: factors include age, gender, and ultrasound features such as tumor location, tumor size, margin, calcification, and abnormal LNs, excluding CLT. The AUC is 0.8929. Model 3: factors include age, gender, and ultrasound features such as tumor location, tumor size, margin, and calcification, excluding CLT and abnormal LNs. The AUC is 0.8556. CCLNM, central cervical lymph node metastasis; CLT, chronic lymphocytic thyroiditis; LNs, lymph nodes; AUC, area under the receiver operating characteristic curve.

Our multivariate analysis revealed that age ≤40 years (OR = 3.796, 95% CI = 2.842, 5.070), male gender (OR = 4.005, 95% CI = 2.858, 5.61), tumor located in the isthmus (OR = 7.578, 95% CI = 4.863, 11.810), macroaxis size (OR = 2.985, 95% CI = 1.581, 5.633) (the cutoff value of tumor macroaxis size was 0.805 cm), ill-defined margin (OR = 3.008, 95% CI = 1.986, 4.556), microcalcification (OR = 2.155, 95% CI = 1.585, 2.929), and abnormal LNs (OR = 13.753, 95% CI = 9.278, 20.385) were independent risk factors for large-volume CCLNM. CLT (OR = 0.248, 95% CI = 0.172, 0.358) was a protective factor. No significant differences were observed for tumor transverse size (OR = 1.087, 95% CI = 0.464, 2.546), anteroposterior size (OR = 1.014, 95% CI = 0.380, 2.705), and irregular shape (OR = 1.045, 95% CI = 0.789, 1.383) (**Table 3**). A receiver operating characteristic (ROC) curve was drawn using the significant factors (**Figure 2**). In addition, ultrasound and pathological results were compared in this study. The sensitivity, specificity, and accuracy of preoperative neck ultrasound for central compartment LNM were 84.42%, 76.1%, and 77.49%, respectively (**Supplementary Table 1**).

## Discussion

PTC is prone to LNM, and the metastasis rate can reach 17%–90% (6, 7). The presence of more than five metastatic LNs is classified as large-volume LNM, which is correlated with a 20% risk of recurrence and is associated with lung metastasis. The 2015 ATA guidelines consider large-volume cervical LNM as a medium-risk factor for recurrence. It is generally accepted that tumor cells spread in a stepwise way: from central to lateral neck regions. Skip metastases that omit the central compartment and spread initially in lateral neck levels only occur in a small number of patients (8). In addition, researchers confirmed that large-volume CCLNM was an independent risk factor of lateral LNM in PTC (9). Moreover, compared with small-volume LNM, it was demonstrated that large-volume LNM is significantly associated with decreased recurrence-free survival (10). At present, the indication of prophylactic central LN dissection without LNM detected by ultrasound remains controversial, and an increasing number of countries are paying attention to whether there is excessive treatment for thyroid cancer. In fact, the diagnostic efficiency of preoperative ultrasound for central compartment LNM is poor. The comparison results of ultrasound and pathology in this study were roughly consistent with previous studies (11). However, preoperative ultrasound and other imaging examinations, such as computed tomography (CT) and magnetic resonance imaging (MRI), are not highly sensitive to central LNM (12, 13). Therefore, investigation of the clinical and ultrasound characteristics of large‐volume LNM metastases is of guiding significance for surgical management. Although there have been a large number of studies on the risk factors associated with LNM in PTC, this study is the first comparative analysis of large-volume LNM versus no LNM.

Comparing the two groups, the results showed that multiple indicators were significantly associated with large-volume CCLNM. The risk degree of independent risk factors was ordered as follows: from high to low, abnormal LNs (OR = 13.753, 95% CI = 9.278, 20.385), tumor located in the isthmus (OR = 7.578, 95% CI = 4.863, 11.810), male gender (OR = 4.005, 95% CI = 2.858, 5.61), age ≤40 years (OR = 3.796, 95% CI = 2.842, 5.070), ill-defined margin (OR = 3.008, 95% CI = 1.986, 4.556), macroaxis size (OR = 2.985, 95% CI = 1.581, 5.633), and microcalcification (OR = 2.155, 95% CI = 1.585, 2.929). CLT (OR = 0.248, 95% CI = 0.172, 0.358) was a protective factor for CCLNM. When one or more of these features appear, it is likely to have been large-volume CCLNM, which is easy to recur, warning the clinic to give appropriate treatment. Moreover, the 2015 ATA guidelines, the 2017 American College of Radiology TI-RADS, and the 2017 European Thyroid Association EU-TIRADS are all based on the ultrasound characteristics of thyroid nodules (4). In addition to the characteristics of nodules, the clinical data of patients, such as age, gender, CLT, and abnormal LNs on ultrasound, were also included in this study.

Previous studies suggest that cystic appearance, microcalcification, or hyperechoic mass in LNs may be important characteristics of LNM in PTC (5). Our study showed that the risk of large-volume CCLNM was increased by 13 times when abnormal LNs were detected by preoperative ultrasound; that is, abnormal LNs were highly indicative of large-volume CCLNM, which is of great significance for the selection of surgical methods.

In the present study, compared with bilateral tumors, isthmus tumors were associated with an increased risk of large-volume CCLNM, which was consistent with previous studies (14, 15). This may be related to the special anatomical location of the isthmus and abundant lymphatic reflux (16).

As is well known, the incidence of thyroid cancer has an obvious gender tendency; the incidence in women is significantly higher than that in men. Current studies have shown that this may be associated with higher estrogen levels in women. However, once the protective mechanism is impaired and the malignant environment associated with progressive thyroid cancer and LN metastasis is exposed, LNM is more likely to occur (6, 7). In this study, the risk of large-volume CCLNM in male patients was 4.005 times higher than that in women (95% CI = 2.858, 5.61), which is consistent with a previous study (17).

There is no consensus regarding the influence of age and LNM on PTC. Liu et al. (18) found that cervical LNM was not related to age, while some studies showed that age ≤45 years was associated with an increased risk of large-volume CCLNM (19, 20). In one study in Japan, young patients (<40 years old) showed more tumor growth than older patients (21). Oh et al. (22) found that age <40 years was one of the main predictive factors for LNM and recurrence. In the present study, younger age (≤40 years) was associated with an increased risk of large-volume CCLNM. Therefore, it is necessary to pay attention to the condition of LNM when planning treatment for patients aged ≤40 years.

Numerous studies have shown that ill-defined margin and microcalcification both reflect stronger proliferation and invasion of malignant cells; thus, the risk of LNM is higher (19).

In the present study, we showed that the macroaxis size of the tumor is an independent risk factor for large-volume CCLNM. The risk of large-volume CCLNM increased 1.985 times with a 1-unit increase in the macroaxis size, and the cutoff value was 0.805 cm, indicating that nodules with a macroaxis size of >0.8 cm require special attention.

The relationship between CLT and PTC has always been controversial (23, 24). Our study suggests that CLT is a protective factor for large-volume CCLNM; this may be related to the autoimmunity of CLT patients (25). The autoimmunization process associated with CLT is associated with immune factors and immune cells, which regulate the tumor microenvironment, enhance the anti-tumor immune response, and limit the growth and metastasis of tumor cells. When combined with CLT, the local tissue is infiltrated by a large number of immune cells, which produce antibodies, killing tumor cells and preventing malignant cells from spreading (26).

The predictive model 1 was established by using the statistically significant factors in multivariate analysis (age, gender, CLT, and ultrasound features like tumor location, tumor size, tumor margin, calcification, and abnormal LNs). The area under the ROC curve (AUC) was 0.9008. When using model 2 (excluding CLT), the AUC value was 0.8929. The AUC of model 3 (excluding CLT and abnormal LNs) was 0.8556. These results show that model 1 is effective in predicting large-volume CCLNM.

The limitations of this study include the following. This study was a retrospective analysis, which was limited to PTC patients diagnosed in one hospital. This may lead to bias. In addition, this study only compared large-volume CCLNM and no CCLNM, while the factors related to one to five LN metastases in central cervical and bilateral cervical LNM were not studied. Finally, we did not use the significant factors to construct a predictive scoring system for large-volume CCLNM.

## Conclusion

Taken together, the following factors are positively related to large-volume CCLNM in PTC patients: age ≤40 years, male sex, tumor macroaxis size >0.8 cm, tumor located in the isthmus, ill-defined margin, microcalcification, and abnormal LNs. These factors are also associated with an increased risk of recurrence, suggesting that more aggressive surgical approaches such as prophylactic central compartment LN dissection may be necessary. Finally, CLT can be regarded as a protective factor.

## Data availability statement

The original contributions presented in the study are included in the article/**Supplementary material**. Further inquiries can be directed to the corresponding authors.

## Ethics statement

The studies involving human participants were reviewed and approved by the ethics committee of Peking Union Medical College Hospital. The patients/participants provided their written informed consent to participate in this study.

## Author contributions

KL and YL conceived and designed the study. LT and JJ were the major contributors in performing the analysis, writing the manuscript, and preparing the figures and tables. GS and KL participated in the study design and edited the manuscript. GS, YL, and KL participated in the verification of data, image quality verification, selection, and collection of samples. All authors contributed to the article and approved the submitted version.

## Funding

This study was funded by the National Natural Science Foundation of China (No. 81873902), the National Natural Science Foundation of China (NSFC) (award number: 82171968), and the Chinese Academy of Medical Sciences Innovation Fund for Medical Sciences (CIFMS) (No. 2020-I2M-C&T-B-039).

## Conflict of interest

The authors declare that the research was conducted in the absence of any commercial or financial relationships that could be construed as a potential conflict of interest.

The reviewer YW declared a shared affiliation with the authors to the handling editor at the time of review.

## Publisher’s note

All claims expressed in this article are solely those of the authors and do not necessarily represent those of their affiliated organizations, or those of the publisher, the editors and the reviewers. Any product that may be evaluated in this article, or claim that may be made by its manufacturer, is not guaranteed or endorsed by the publisher.
