# Supplementary material for: Related factor analysis for predicting large-volume central cervical lymph node metastasis in papillary thyroid carcinoma
Source: Front Endocrinol (Lausanne). 2022 Aug 15;13:935559. doi: 10.3389/fendo.2022.935559 (PMC9423095; doi:10.3389/fendo.2022.935559)
Supplement: Supplementary file 1 [file Table_1.docx]

**Supplementary Table 1** LNM comparison of ultrasound and pathology

| True positive | False positive | True negative | False negative | Sensitivity | Specificity | Accuracy |
| --- | --- | --- | --- | --- | --- | --- |
| 65 | 92 | 293 | 12 | 0.8442 | 0.761 | 0.7749 |
